# Reverse wrap-up effects by reading scenario, boundary salience, and word position: Novel evidence from eye movements in natural Chinese reading

**DOI:** 10.3758/s13423-025-02766-7

**Published:** 2026-02-18

**Authors:** Yushu Wu, Chunyu Kit

**Affiliations:** Department of Linguistics and Translation, City University of Hong Kong, 83 Tat Chee Avenue, Kowloon, Hong Kong SAR China

**Keywords:** Boundary salience, Chinese reading, Eye movements, Word position, Wrap-up effects, Natural reading

## Abstract

This study investigates how wrap-up effects—sentence-final words incur heavier processing loads than sentence-internal words—manifest in natural reading of unspaced, logographic Chinese scripts. We leveraged a large-scale naturalistic reading corpus (subjects: 98; word tokens: more than 1 million by 300 individual sentences and seven passages), employed multifactorial analyses by (generalized) linear mixed-effects models, and compared four eye-movement differences (i.e., gaze duration, total reading time, skipping probability and regression-in probability) between sentence-internal and sentence-final words. The results demonstrated robust reversal of traditional wrap-up effects: sentence-final words required significantly *less* processing effort than sentence-internal words, such as shorter durations, higher skipping rates and lower regression-in probabilities. Further, this reversal was modulated by reading scenarios (sentence vs. passage), boundary salience (period- vs. comma-bounded), and wrap-up positions (pre-critical, critical, and spill-over). Notably, sentence-final words were processed more rapidly when associated with characteristics such as fewer stroke counts, shorter length, higher frequency, function-word status, or progress further into page at the late processing stage. Challenging classic models that attribute inflated time at clause/sentence boundaries to semantic integration, we postulate punctuation’s dual role in unspaced language processing: visual cues for word segmentation/recognition and semantic cues for integration jointly optimize language comprehension.

## Introduction

The term “wrap-up effect” refers to a well-documented phenomenon wherein readers allocate disproportionately greater cognitive resources to processing words at syntactic boundaries (e.g., clause or sentence endings) compared with internal positions. This manifests behaviourally as prolonged inspection times, frequent regressions, and extended saccades at sentence- or clause-final words (Just & Carpenter, 1980; Rayner et al., 2000). Historically, such effects were often treated as methodological noise in controlled experiments, leading researchers to exclude boundary-region data. However, recent work reframes wrap-up effects as critical windows into higher-order comprehension processes, including discourse integration and ambiguity resolution (Andrews & Veldre, 2021; Meister et al., 2022).

### Manifestation of wrap-up effects

Early self-paced reading experiments first documented boundary-specific time inflation, with readers exhibiting longer pauses and response times at sentence endings (Aaronson & Scarborough, 1976; Mitchell & Green, 1978). Subsequent eye-tracking research expanded these findings, identifying distinct early- and late-stage eye-movement measures of wrap-up effects:

1. Early measures (e.g., first fixation duration) reflect immediate lexical access modulated by boundary salience (Hirotani et al., 2006).
2. Late measures (e.g., go-past time, regression probability) index integrative processes such as resolving referential ambiguities or updating discourse models (Rayner et al., 2000; Warren et al., 2009).

Event-related potential (ERP) studies link wrap-up effects to enhanced N400 amplitudes at sentence-final words, reflecting heightened semantic integration demands (Hagoort, 2003). However, recent meta-analyses caution against attributing wrap-up solely to discrete ERP components, as syntactic (P600) and semantic (N400) processes interact dynamically during boundary processing (Stowe et al., 2018). Cross-linguistic studies further demonstrated the robustness of wrap-up effects. In English, clause-final nouns elicited prolonged gaze durations and regressive saccades (Rayner et al., 2000), while Dutch corpus analyses revealed boundary-driven predictability gradients (Kuperman et al., 2010). Further, clause wrap-up was detected in Chinese syntactically ambiguous sentence reading in Luo et al.’s (2013) gaze contingent preview paradigm. Nevertheless, null wrap-up effects were captured in Japanese reading (Asahara, 2018), suggesting orthography-specific segmentation strategies modulate wrap-up dynamics. More recently, reverse wrap-up effects from corpus-based analyses (Wu, 2024) or null effects from controlled experiments (Xue et al., 2025) were observed in Chinese sentence reading.

### Modulating factors of wrap-up effects

Punctuation marks systematically amplify wrap-up magnitudes (periods > commas > unmarked) by signalling hierarchical discourse boundaries (Hirotani et al., 2006; Warren et al., 2009). Periods, denoting topic closure, elicit prolonged spill-over fixations as readers consolidate episodic representations, whereas commas prompt local phrase-boundary updates (Andrews & Veldre, 2021). Positional effects further dissociate pre-critical (predictability-driven) and spill-over (coherence-building) processing (Kuperman et al., 2010). In addition, aging studies reveal divergent strategies: younger adults exhibit classic sentence-final time inflation, while older readers redistribute processing effort to mid-sentence positions (Payne & Stine-Morrow, 2012, 2014; Stine-Morrow et al., 2010; Tiffin-Richards & Schroeder, 2018). Cross-linguistic comparisons highlight script-specific mechanisms—Chinese readers leverage punctuation as parafoveal cues (Li & Pollatsek, 2020), whereas Japanese readers show reduced boundary effects due to morpho-syntactic demarcation (Asahara, 2018).

### Motivations, research questions, and hypotheses

Based on the extant literature, we discover several issues at stake. Firstly, studies of Chinese readers performing sentence wrap-up have not given prominence. Secondly, controversial studies exist over the direction of wrap-up effects, for some disclose greater time on sentence-final words, while others show otherwise. Thirdly, the interplay of wrap-up effects with other linguistic variables is underexplored. Given these unsettled challenges, we are motivated to investigate wrap-up effects in natural Chinese reading, with a particular emphasis on comparison of sentence and passage reading. We formulate five research questions (RQs):

1. Is there a significant wrap-up effect in Chinese reading?
2. Do the sentence-final words require increased reading time compared with sentence-internal ones?
3. Is there a significant difference in the wrap-up effects between sentence and passage reading?
4. Can word properties (e.g., length, frequency, stroke count, word type, part-of-speech, and radical type) significantly impact eye movements of wrap-up process?
5. Can word position significantly impact eye movements of wrap-up words reading?
6. Can boundary salience (comma-marked vs. period-marked) significantly impact eye movements of wrap-up words reading?
7. For the final words, in which wrap-up position (pre-critical, critical, and spill-over), do the wrap-up effects have the most pronounced indication?

In response to the questions above, we predict possible outcomes as per the established studies: (1) Following the classic wrap-up effect observed in alphabetic reading, Chinese reading may exhibit robust wrap-up effect wherein distinct eye-movement differences can be found in internal and final words. (2) compared with internal words, processing final words may require more cognitive effort, such as longer durations, lower skipping rates, and greater likelihood of regressions. (3) The wrap-up effect can be more pronounced in sentence reading compared with passage reading, with the latter maintaining more efficient process. (4–5) Content words might need more reading time than function words, but we leave the effects of part-of-speech, radical type, and word position open. (6) Compared with clause wrap-up, sentence wrap-up may have stronger manifestation. (7) Processing words in the pre-critical or critical region requires reduced reading time than processing spill-over words, for the latter may serve as a spill-over region responsible for ultimate integration for the previous sentence *N* and novel information retrieval for the subsequent sentence *N* + 1.

## Method

### Data import and description

**Fig. 1:**
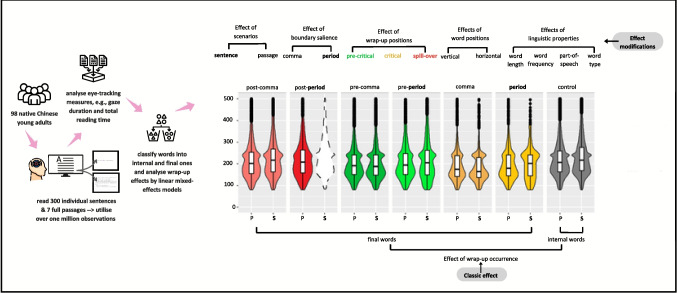
An overview of the methodological process. (Colour figure online)

To highlight several main components of the Method section, a schematic overview is illustrated in Fig. 1, including corpus description, data classification, and data analysis. Following a corpus-based approach (Asahara, 2018; Kennedy & Pynte, 2005; Kuperman et al., 2010; Meister et al., 2022), this study targets at constructing statistical models based on datapoints from an eye-movement corpus and comparing reading behaviours of individual sentences and paragraphs. We leveraged a newly released natural reading database, *Hong Kong Corpus of Sentence and Passage Reading* (HKC; Wu & Kit, 2023). The corpus selected sentences with lowest average entropy for ensuring noticeable contextual surprisal/inconsistency to its most. We employed automatic word segmentation and manual corrections based on the national standard GB/T 13715–92 (1992) for identifying word-based interest areas. It records 98 young adults (mean age: 26) reading simplified Chinese scripts including 300 single sentences and seven full passages of 5,250- and 4,967-word tokens. To avoid intervening effects of return-sweeps, line wrap-up, and line start-up, we did data trimming and filtered out line-head words, line-tail words, and sentence-head words in single-sentence reading (5.53% of the total), which left us with 949,132 data points and 28 eye-movement measures.

### Data preparation

We extracted four commonly used eye-movement measurements from the imported corpus as dependent variables (response variables): gaze duration (GD: dwell time in the first-run reading; *M* = 240.09, *SD* = 123.29), total reading time (TRT; *M* = 351.59, *SD* = 265.92), probability of skipping a word in the first run reading (PS; *M* = 67%), and probability of being regressed back into a word (RI; *M* = 35%), in addition to type of reading scenarios (sentence reading vs. passage reading).

Afterwards, we created three independent variables (occurrence, wrap-up position, and boundary salience) related to examining sentence wrap-up. First, we formulated a logical variable, *wrap-up occurrence*, distinguishing internal and final words, which has two opposite levels: “yes”, if at the boundary-region (i.e., sentence-final or clause-final position); “no”, if at other positions. Second, we formulated a categorical variable, *wrap-up position*, used for denoting word positions of final words with three levels (“pre-critical”, if before the period/comma; “critical”, if at the period/comma; “spill-over”, if after the period/comma). Third, we made another categorical variable, *boundary salience*, used for discriminating final words ended by two punctuation marks: “comma” if final words are marked by clause punctuation mark “,”; “period”, if final words are marked by sentence punctuation mark “。”. It should be noted that the “spill-over” level of the variable wrap-up position” does not exist in single sentence reading, as there is no more lexical information after the sentence period. Meanwhile, the spill-over word of sentence *N* is equivalent to the headword of sentence *N* + 1 in passage reading.

To further examine linguistic factors, we expanded the HKC corpus with annotations of *word type, vertical/horizontal word positions, part-of-speech* (POS), and *type of radical structure* for each character by machine learning (*jieba* and *pandas *packages; McKinney & the Pandas Development Team, 2023; Sun, 2023). *Word type* distinguishes content words, function words, and pronouns, with different proportional distribution across sentence-ending words (content words: 47.1%; function words: 11.6%; pronouns: 2.9%; punctuation: 38.4%), while *vertical word position* (from top- to bottom-edge, 0 to 1) and *horizontal word position* (from left- to right-edge, 0 to 1) measure the relative positions of each word to the page. The *part-of-speech* (POS) denotes grammatical class of a word such as noun, verb, or adjective, and *radical structure *categorises a Chinese character into six positional combinations of radicals: Left–Right (LR), Up-Down (UD), Left–Right-Bottom (LRB), Half Circle (HCI), Circle (CIR), and Single (SG), e.g., “明” brightness, “边” side, “草” grass, “回” return, “区” area, and “开” open. The proportional distributions of part-of-speech (verbs: 17%; nouns: 12.7; prepositions: 4.1%; adverbs: 3.5%; adjectives: 3.1%; pronouns: 2.9%; numeral: 2.6%; conjunctions: 1.4%; punctuations: 38.4%) and radical structure (LR: 22.9%; HCI: 2%; SG: 16.1%; UD: 14.9%; LRB: 2.6%; CIR: 1%; punctuation: 40.1%) for sentence-ending words are also presented here.

### Data analysis

To enhance the statistical efficacy of generalized linear mixed-effects models ((G)LMMs) we employed for data analysis, three additional linguistic variables were borrowed from the *Chinese Lexical Database* (Sun et al., 2018), a corpus that assimilates hundreds of linguistics properties, and annotated to each word in our target corpus. Subsequently, we delineated the variables into three categories A, B, and C:

A. Fixed effects:

a Independent variables:
  i. scenario and wrap-up occurrence.
  ii. scenario, boundary salience, and wrap-up position.
b Covariates:
  i. word length, word stroke count (i.e., the total number of strokes in a word), word frequency.
  ii. POS tag, word type, type of radical structure of first character (C1Structure), type of radical structure of second character (C2Structure).
  iii. vertical word position, horizontal word position.

B. Random effects: items and subjects.

C. Dependent variables for each (G)LMM: GD, TRT, PS, or RI.

Regarding effects of wrap-up occurrence, reading scenario, part-of-speech, word type, and word positions (for resolving the Research Questions 1–5), we built four (G)LMMs, with each model first consolidated by all random effects and updated by deducting them individually. Given the problematic convergence of random slopes, fixed slopes were used with random intercepts across subjects and items. Among those models, we chose the models that attained the lowest value of the Akaike information criterion and then incorporated all fixed effects (under subcategories **a-i** and **b**) into it. The numeric variables under **bi** were centred to enhance regression performance, the maximum likelihood estimation employed to address the missing data (referred to as “NA”), and the expectation–maximization algorithm chosen to update the parameters. After implementing a backward selection process, the model dropped unnecessary variables that either did not contribute to or had variance inflation factors (VIF) of five or more to avoid collinearity.

Regarding the modulating factors of wrap-up effect such as boundary salience and wrap-up position (for resolving the research questions 6–7), we altered the independent variable from the subcategory **a-i** to **a-ii**, while leaving the other fixed effects and fitting procedure intact. The purpose of such alteration is to do further comparisons for wrap-up effect clarifications and to capture the reading performance of wrapping up by boundary salience and wrap-up position.

## Results

### The existence and direction of wrap-up effects

**Fig. 2:**
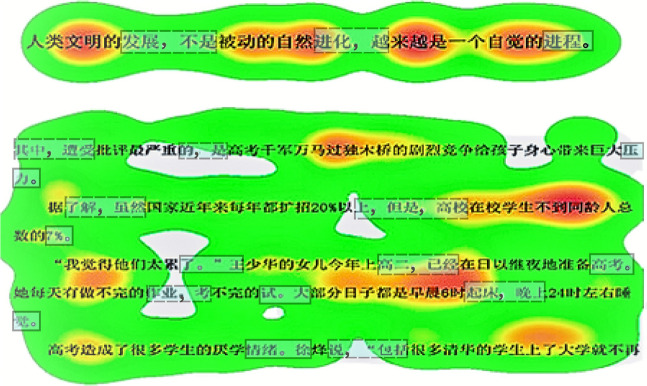
Heatmap for example sentence and paragraph reading across subjects. *Note*. Both sentence- and clause-final regions are roughly framed by rectangles. The heatmap records the dynamics of reading time from 0 to 1000 ms, varying in white, green, and orange colour. (Colour figure online)

For a rough observation for internal and final words prior to furthering model results, Fig. 2 provides a heatmap illustrating the temporal distribution across sentence-internal and—final words in example sentence and passage reading (see top- and bottom-sections for the scenario comparison). By specifically framing final-word regions, we take stock of the majority trend for internal words and spot them in darker colour, which signals *more* inspection time on internal words compared with final words. Notwithstanding coherently observed time inflation in internal words during both sentence and passage reading, we must verify its reliability by employing stricter statistic tests.

### Modulating factors on wrap-up effects

#### Reading scenario, word properties, and word positions on wrap-up effects

Overall indication of wrap-up occurrence by key factors reading scenario, linguistic properties and word positions is displayed in the Table 1, and four (G)LMMs jointly demonstrated a reverse pattern for sentence wrap-up in Chinese reading, as opposed to the classic one in English reading (Just & Carpenter, 1980). For brevity, Table 1 showcases partial results, with a complete report stored in the Open Science Framework (see Table 1*Wrap-up effects* at https://osf.io/yxkz9/?view_only=11786e2d29a84d4d811d191ebf31b8e4).

**Table 1:** Effects of wrap-up occurrence, reading scenario, word type, word positions, part-of-speech, and radical structure in the (G)LMMs

	GD		TRT		IA_SKIP		IA_REGRESSION_IN	
Predictors	*Estimates*	*std. Error*	*Estimates*	*std. Error*	*Odds Ratios*	*std. Error*	*Odds Ratios*	*std. Error*
(Intercept)	225.009 ^***^	4.473	344.903 ^***^	20.517	3.311 ^***^	*0.168*	1.028	0.065
Scenario [S]	25.990 ^***^	0.68	83.225 ^***^	0.99	0.347 ^***^	*0.002*	1.285 ^***^	0.012
W occur [yes]	− 7.434 ^***^	7.197	15.919	9.676	1.762 ^***^	*0.021*	1.016	0.019
Vertical word position	8.327 ^***^	1.444	− 0.485	1.884	0.628 ^***^	*0.009*	0.733 ^***^	0.016
Horizontal word position	11.728 ^***^	1.009	− 21.207 ^***^	1.45	0.847 ^***^	*0.009*	0.166 ^***^	0.002
Len scaled	25.973 ^***^	2.202	55.702 ^***^	5.795	0.470 ^***^	*0.012*	1.045	0.05
Fre log	− 8.262 ^***^	0.745	− 19.077 ^***^	1.956	1.137 ^***^	*0.013*	1.001	0.022
Stro scaled	0.26	0.161	0.562	0.42	0.988 ^***^	*0.002*	0.989 ^*^	0.004
Scenario [S] * W occur[yes]	− 14.632 ^***^	1.37	− 76.926 ^***^	1.789	1.446 ^***^	*0.021*	0.691 ^***^	0.015
Word Type [function]	16.666	18.378	24.788	45.509	*/*	*/*	1.052	0.06
Word Type [pronoun]	− 3.401	5.873	− 2.928	14.788	*/*	*/*	0.949	0.132
W occur [yes] * Word Type[function]	− 4.186	7.762	** − 24.537**	9.777	*/*	*/*	0.994	0.032
W occur [yes] * Word Type[pronoun]	15.410 ^*^	5.248	28.279 ^***^	6.842	*/*	*/*	1.882 ^***^	0.097
W occur [yes] * POS Tag[n]	− 5.463	3.403	− 17.564 ^***^	4.606	*/*	*/*	/	/
W occur [yes] * POS Tag[ng]	− 4.551	9.61	26.985	15.299	*/*	*/*	/	/
W occur [yes] * POS Tag[nz]	− 15.49	19.568	** − 58.424**	26.065	/	/	/	/
W occur [yes] * POS Tag[p]	5.499	7.846	**20.913**	9.904	/	/	/	/
W occur [yes] * POS Tag[q]	− 9.168	9.382	18.1	12.793	/	/	/	/
W occur [yes] * POS Tag[s]	− 51.376	31.867	− 68.558	78.545	/	/	/	/
W occur [yes] * POS Tag[u]	4.28	15.7	65.533 ^*^	22.802	/	/	/	/
W occur [yes] * POS Tag[ug]	− 10.446	13.385	51.142 ^*^	16.247	/	/	/	/
W occur [yes] * POS Tag[ul]	− 11.235	16.913	** − 47.565**	22.175	/	/	/	/
W occur [yes] * POS Tag[v]	− 3.348	3.406	** − 9.767**	4.644	/	/	/	/
W occur [yes] * POS Tag[vn]	** − 11.039.**	5.588	− 23.447 ^*^	7.772	/	/	/	/
W occur [yes] *C1Structure [LR]	− 1.182	4.94	− 19.928	15.039	/	/	/	/
W occur [yes] *C1Structure [LRB]	6.569	5.995	− 41.815 ^*^	14.825	/	/	/	/
W occur [yes] *C1Structure [SG]	3.833	5.07	− 37.969 ^*^	12.907	/	/	/	/
W occur [yes] *C1Structure [UD]	− 1.282	5.016	− 21.224	12.834	/	/	/	/
W occur [yes] *C2Structure [HCI]	** − 11.068.**	5.491	9.191	14.388	/	/	/	/
W occur [yes] *C2Structure [LR]	− 5.211	4.782	9.122	15.733	/	/	/	/
**Random Effects**								
σ^2^	15,614.41		47,502.04		3.29		3.29	
τ_00_	395.82 _Item_		3569.81		0.16 _Item_		0.58 _Item_	
	1041.13 _Subject_		2462.33		0.20 _Subject_		0.20 _Subject_	
ICC	0.08		0.11		0.1		0.19	
*N*	98 _Subject_		98 _Subject_		98 _Subject_		98 _Subject_	
	1706 _Item_		1706 _Item_		2115 _Item_		2115 _Item_	
Observations	319,953		504,927		949,132		487,138	
Marginal *R*^2^/Conditional *R*^2^	0.022/0.104		0.046/0.154		0.159/0.241		0.062/0.242	
Formulas	GD ~ Scenario * W_occur + Word_Type * W_occur + POS_Tag * W_occur + len.scaled vertical_word_position + Fre.log + stro.scaled + C1Structure * W_occur + C2Structure * W_occur + horizontal_word_position + (1 | Subject) + (1 | Item)		TRT ~ Scenario * W_occur + Word_Type * W_occur + POS_Tag * W_occur + len.scaled vertical_word_position + Fre.log + stro.scaled + C1Structure * W_occur + C2Structure * W_occur + horizontal_word_position + (1 | Subject) + (1 | Item)		PS ~ Scenario * W_occur + vertical_word_position + horizontal_word_position + len.scaled + Fre.log + stro.scaled + (1 | Subject) + (1 | Item)		RI ~ Scenario * W_occur + Word_Type * W_occur + vertical_word_position + horizontal_word_position + len.scaled + Fre.log + stro.scaled + (1 | Subject) + (1 | Item)	

1. By Bonferroni correction, we changed alpha from.05 to.0125 (critical t/z value = 0.05/4), and updated significance level with the symbol^**.**^ standing for *p* < 0.1, * for *p* < 0.05, ** for *p* < 0.01, and *** for *p* < 0.001. 2. Regarding POS tags, reference is set as Adjective, and c = conjunction; d = adverb; j = abbreviation; n = noun; nz = other proper noun; p = preposition; q = measure word; s = locative; t = time word; u = particle; ug = "*guo*" particle (past tense marker); ud = "*di*" Particle (adverbial marker); ul = "*le*" Particle (completion marker); v = verb; vn = verbal noun. 3. Regarding radical structure, reference is set as Circle (CI), and Left–Right = LR; Left–Right-Bottom = LRB; Up-Down = UD; Half Circle = HCI; Single = SG. After the models were joined by word type and POS, wrap-up occurrence had an inflated VIF; we retained them in the models for richer wrap-up manifestations.

**Fig. 3:**
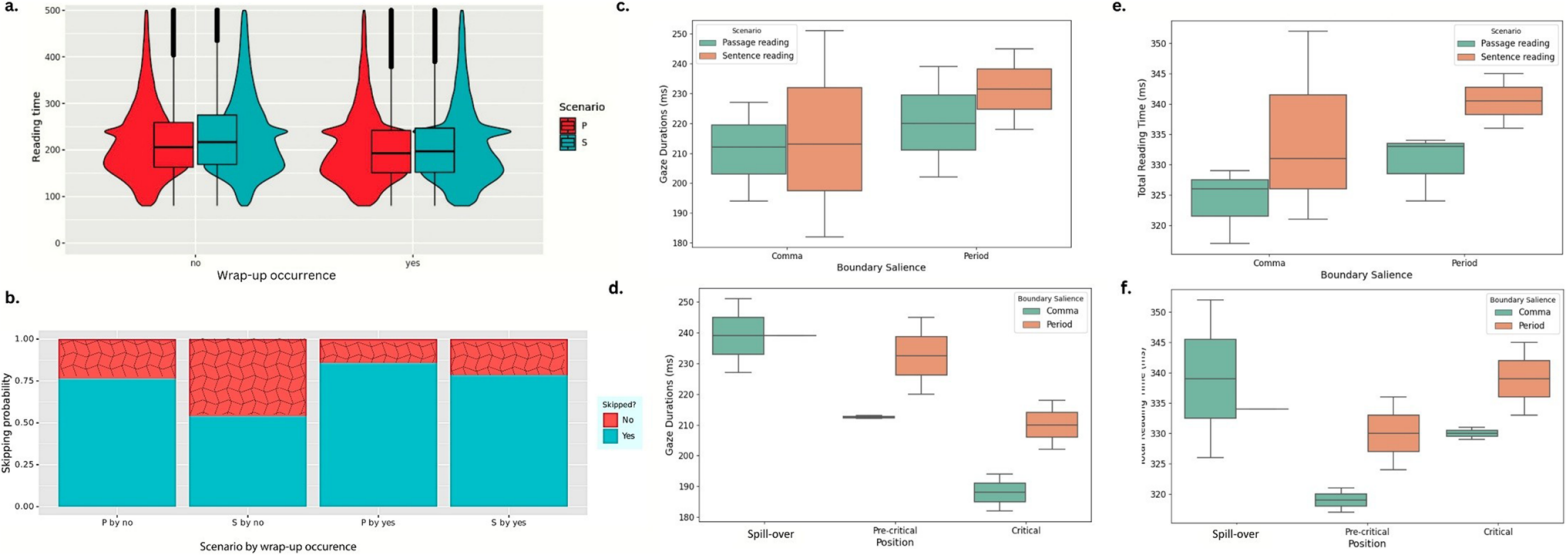
Effects of wrap-up occurrence by reading scenario, boundary salience, and wrap-up positions. *Notes*. Figure 3 consists of six subfigures labelled by a, b, c, d, e, and f. Figure **a**-**b** describe wrap-up effects on gaze durations and skipping probability, with top legend denoting the factor of scenario (P: passage reading; S: sentence reading) and bottom legend denoting the skipping probability (No: no skipping and fixations happen; Yes: skipping happens and no fixations). Figure **c-d** separately visualise the interaction effects, of (1) boundary salience and scenarios, and (2) the interaction effects of boundary salience and wrap-up positions, on gaze durations. Likewise, Figure **e–f** visualise the two interaction effects on total reading time. (Colour figure online)

The main findings of wrap-up effects and the effect modification are summarised in Fig. 3. In Fig. 3a, gaze durations for final words (i.e., the level of occurrence: *yes*) prominently dropped (β = − 7.43, *SE* = 7.20, *p* < 0.001), compared with the time spent on internal words (i.e., the level of occurrence: *no*). Further, no robust differences were revealed in total reading time (β = 15.92, *SE* = 9.68 *p* = 0.09), manifesting null wrap-up effects. Figure 3b shows distinct differences of skipping probability, with the odds of the skipping probability twice higher for final words than internal ones (*OR* = 1.76, *SE* = 0.02, *p* < 0.001).

To examine the interaction effect of reading scenario (sentence reading vs. passage reading) and occurrence on gaze durations in a pairwise way, we carried out post hoc multiple pairwise comparisons (see Table 2 for details), by adopting the Tukey’s adjustment and setting justified Type I error rates (α < 0.001). The greatest wrap-up distinction occurred in individual sentence (β = 23.15, *SE* = 0.70, *z* = 33.054, *p* < 0.001): sentence-internal words had 11 ~ 23 ms time inflation in both sentence and passage reading. Besides, sentence reading kept having greater wrap-up magnitudes than passage reading by 7 ~ 19 ms, whenever reading internal or final words.

**Table 2:** Interactional effects of scenario and occurrence on gaze durations

Scenario by Occurrence	β	*SE*	*z*	*p*
P no—S no	− 19.42	0.451	− 43.03	<.001***
P no—P yes	11.05	0.84	13.146	<.001***
S no—S yes	23.15	0.70	33.054	<.001***
P yes—S yes	− 7.32	0.994	− 7.368	<.001***

1. *p < 0.05, **p < 0.01, ***p < 0.001. 2. S = sentence reading; P = passage reading

Robust modulations of linguistic properties (e.g., word length, word frequency, word stroke count, word type, part-of-speech, and type of radical structure) and horizontal/vertical word positions on wrap-up magnitudes are confirmed. First, final words that had shorter length, less stroke count, or higher frequency suggested less pronounced gaze duration (β = 10.99, *SE* = 1.45,* p* < 0.001; β = 0.47, *SE* = 0.13,* p* < 0.001; β = − 7.87, *SE* = 0.66,* p* < 0.001). Second, sentence-final content words needed shorter time than pronouns (TRT: β = 18.78, *SE* = 3.51, *p* < 0.001), but longer time than function words at the late stage (TRT: β = − 61.10, *SE* = 7.09,* p* < 0.001). Third, part-of-speech manifested saliently wide influence on wrap-up suggestion: reading adjective final words required less total reading time than adverb/particle-marked counterparts but more time than noun/verb counterparts. Interestingly, types of radical structure projected robust modulation on wrap-up magnitudes: sentence-final words featuring single or left–right-bottom structure had significantly shorter total reading time than sentence-internal words (β = − 41.815, *SE* = 14.825, *p* < 0.05; β = − 37.969, *SE* = 12.907, *p* < 0.05). Finally, the further readers progressed into the text (toward the right or bottom of the page), the greater time at the early stage (GD = 12.25, *SE* = 0.72, *p* < 0.001; β = 6.87, *SE* = 1.05, *p* < 0.001) but less time at the late stage (TRT: β = − 0.62, *SE* = 1.04, *p* = 0.55; β = − 10.02, *SE* = 0.80, *p* < 0.001).

#### Boundary salience and wrap-up positions on wrap-up effects

Building on the results of pronounced differences in wrap-up effects, we showcase Table 3 for observing the mean gaze durations and total reading time (with standard deviations included) grouped by modulating factors such as boundary salience, wrap-up positions, and reading scenarios. As a general observation, we detect subtle temporal differences across all conditions. For example, comma-bounded words in the spill-over region required shorter gaze durations than their counterparts in passage reading (post-comma: 212 ms; post-period: 220 ms), with larger advantage occurring in sentence reading and similar advantage in the spill-over and critical regions.

**Table 3:** Means and standard errors of GD and TRT by boundary salience, wrap-up positions, and reading scenarios

Boundary salience	Wrap-up position			Scenarios	Gaze duration		Total reading time	
					Mean	*SE*	Mean	*SE*
Comma		Spill-over	Passage reading		227	1.17	326	0.61
Comma		Spill-over	Sentence reading		251	1.05	352	0.96
Period		Spill-over	Passage reading		239	1.88	334	1.02
Period		Spill-over	Sentence reading		/	/	/	/
Comma		Pre-critical	Passage reading		212	0.94	317	0.60
Comma		Pre-critical	Sentence reading		213	0.78	321	0.79
Period		Pre-critical	Passage reading		220	1.41	324	0.85
Period		Pre-critical	Sentence reading		245	1.42	336	0.80
Comma		Critical	Passage reading		194	1.15	329	0.35
Comma		Critical	Sentence reading		182	1.06	331	0.28
Period		Critical	Passage reading		202	1.99	333	0.43
Period		Critical	Sentence reading		218	4.48	345	0.12

Table 4 presents the statistical results of our linear mixed-effects models, supporting prominent interaction effects of boundary salience and wrap-up position on both gaze duration (β = 22.70, *SE* = 2.45, *t* = 9.27, *p* < 0.001) and total reading time (β = 12.83, *SE* = 1.92, *t* = 6.67, *p* < 0.001) across six conditions (boundary salience: comma/period; wrap-up positions: pre-critical/critical/spill-over). Regarding the wrapping-up modulated by boundary salience (see Fig. 3c–f for more details), comma-marked wrap-up kept weaker manifestation than period-marked one. Regarding the impact of wrap-up position (see Fig. 3d and f), spill-over words demanded for greatest reading time, and critical words the least in passage reading (pre-critical: 212 ms, critical: 194 ms, and spill-over: 227 ms). Alike pattern was also manifested in sentence reading (pre-critical: 245 ms and critical: 218 ms). It is noteworthy that the inflated time also took place in the beginning of clauses/sentences in Chinese passage reading.

**Table 4:** Modulating factors on wrap-up effects

Gaze duration						Total reading time				
Fixed effects	β	*SE*	*t*	*p*	VIF	β	*SE*	*t*	*p*	VIF
Intercept	212.04	3.91	54.18	0.001 ***	/	34.5	3.71	90.1	< 0.001 ***	/
Boundary salience: Position	22.70	2.45	9.27	< 0.001 ***	1.35	12.83	1.92	6.67	< 0.001 ***	1.09
Boundary salience: Scenario	17.89	1.82	9.84	< 0.001 ***	1.71	9.67	1.41	6.86	< 0.001 ***	1.13
Length	16.33	2.07	7.7	< 0.001 ***	1.40	15.98	3.24	4.93	< 0.001 ***	1.40
Frequency	− 6.55	0.89	− 7.9	< 0.001 ***	1.26	− 6.18	1.42	− 4.3	< 0.001 ***	1.23
Word stroke count	0.38	0.17	2.24	0.0254 *	1.29	− 0.23	0.27	− 0.86	0.39	1.31
Position: Scenario	− 17.18	2.47	− 6.96	< 0.001 ***	2.11	/	/	/	/	/

*Note.* *p < 0.05, **p < 0.01, ***p < 0.001

## General discussion

For a retrospection on our hypotheses, the present study yields several interesting findings, and some challenge classical perspectives on wrap-up effects in reading. First, we observed significantly *reduced* fixation durations at sentence- and clause-final words in Chinese natural reading—a reversal of the canonical wrap-up effect. Second, wrap-up magnitudes revealed more pronounced in individual sentence reading than passage reading. Third, part-of-speech, word position, boundary salience (periods vs. commas), wrap-up position (pre-critical, critical, spill-over regions) modulated wrap-up effects, with maximal durations occurring at spill-over words bounded by periods, echoing our predictions. Moreover, words with low frequency, greater length/word stroke count, or at right- or bottom-edge word positions triggered time inflation at the early stage.

Unlike spaced reading of alphabetic scripts (e.g., English) or morpho-syntactically demarcated languages (e.g., Japanese), Chinese reading defaults unspaced scripts and has no direct word-spacing markers to help readers detect word boundaries. Such “deficiency” encourages variant oculomotor strategies to dynamically segment unseparated character strings while simultaneously identifying words—a process theorized in the *Chinese Reading Model* (Li & Pollatsek, 2020) as a competitive activation mechanism. Punctuation marks, particularly periods and commas, likely serve as salient parafoveal segmentation cues (Ren & Yang, 2010), enabling readers to pre-process boundary-adjacent words during parafoveal preview and thereby attenuate foveal processing time. This explanation aligns with the evidence that readers of unspaced Thai script identify misaligned vowels, relative letter frequency, and tone marks to facilitate word segmentation (Li et al., 2022), effectively "front-loading" cognitive work to the parafovea and diminishing the need for prolonged wrap-up processing.

Our observed temporal reduction at clause/sentence boundaries resists direct mapping onto classic accounts for normal wrap-up effects (Hirotani et al., 2006; Just & Carpenter, 1984; Warren et al., 2009). As per the account of Just and Carpenter (1984), increased reading time in sentence-final words involved certain process for untangling inconsistent meanings. Further, Hirotani et al. (2006) posited prosodic hesitation at clause boundaries for sharpening focus on detecting inconsistencies in the prior text, and “long wrap-up times are attributed to the need to complete extra semantic and discourse updating work” (p. 439). Warren et al. (2009) postulated more complex processes that integrated oculomotor hesitation mechanism and implicit prosody (Hirotani et al., 2006). Kuperman et al. (2010) regarded sentence-final marks as visual reminders for strategic resting and semantic sorting within the local clause. Stowe et al. (2018) combed the above theories into six categories: (1) syntactic representation based on clause boundaries (2) intra- or intersentence information integration, (3) solving semantic inconsistencies, (4) perceptual and prosodic accounts of sentence-final effects, (5) fixing the unacceptability at ends of the clause wrap-up, or (6) a combination thereof. Reconciling normal/reverse wrap-up effects in spaced and unspaced reading, we contextualize *punctuation’s* *dual role*, visual and semantic cues, to optimise language comprehension. The dual role may function with distinct priority in spaced and unspaced reading:

1. In spaced reading, readers anchor spacing for word identification and mainly rely on period/comma for intersentence/clause information updating, resulting in time inflation for strategic pause and semantic synthesis, which aligns with a pay-later or incremental nature.
2. In unspaced reading, readers initially regard period/comma as visual cues that greatly favour parafoveal word segmentation, overshadowing expected time inflation for semantic integration and giving rise to reverse wrap-up effects at the early processing stage.

We also observed clashing wrap-up findings in Chinese sentence processing. Prior studies identify normal wrap-up patterns in reading sentences ended by “*de”* auxiliaries (Zhang & Li, 2017) or syntactically ambiguous prosodic boundaries (Luo et al., 2013). More newly emerging controlled experiments (Xue et al., 2025) and corpus-based exploration (Wu, 2024) furnish solid evidence for reverse wrap-up effects. Weighing up all these findings, we attribute these normal wrap-up effects to complexity/ambiguity manipulation on clause/sentence boundaries. According to our postulation of punctuation’s *dual role*, ambiguous contexts amplify punctuation’s semantic role for inter-sentence integration that would trigger time inflation, whereas normal contexts echo punctuation’s prominent role as visual cues for early word segmentation, mitigating expected time inflation at clause/sentence boundaries. Noteworthily, we should be alert of oversimplifying the differences between spaced and unspaced reading, and thus the proposed *dual role* of punctuation serves simply as one of drivers for reverse wrap-up effects. As Xue et al. (2025) explained, *balanced/non-incremental syntax processing* is another driver wherein information distribution over Chinese sentential contexts peaked at whenever word surprisal occurred rather than at the sentence-endings.

The attenuated wrap-up magnitudes in passage reading compared with isolated sentence reading corroborate Just and Carpenter’s (1987) framework, wherein coherent discourse contexts permit incremental text representation construction, reducing per-sentence integration demands. Longer passage length lends more prior contextual support and diminishes surprisal carried in information flow, which buys reading time for sentence-ending wrap-up. Conversely, disjointed sentence reading necessitates repeated reinitialization of mental models, amplifying boundary-specific processing. Regarding the attenuated clause wrap-up than sentence wrap-up, boundary salience exerted differential effects on wrap-up durations. Periods, signalling topic closure, elicited longer spill-over fixations than commas, which denote intratopic pauses. We interpret this through a dual-process lens: First, in the module of closure processing, periods trigger discourse-level integration (e.g., consolidating episodic representations from working memory), whereas commas prompt local phrase-boundary updates. Second, in the module of predictability gradient: spill-over words following periods (sentence *N* + 1 onsets) exhibit lower predictability than pre-critical words (sentence *N* endings), necessitating extended processing to establish new topic coherence, or a likely different cognitive role rather than semantic integration. Nevertheless, further discussion upon such spill-over is beyond this paper’s scope.

### Limitations and future direction

Given that our postulation about reverse wrap-up effects is based on natural Chinese reading mechanisms, we rely more on the nuances of eye movements. That is, naturalistic effects we capture are significant, real, yet small. Building on our results, future studies can employ controlled experiments, preferably with tighter strictly manipulation of confounding variables, distinguish gross distinction of eye movements, and further disentangle the observed effects at the ends of sentences. Comparisons between spaced and unspaced scripts (e.g., English and Thai) at the alphabetic level could stem the word-spacing factor from other low-level visual cues. More underexplored confounders influencing the direction of wrap-up effects can be POS ambiguity, information surprisal, intonations, reading mode (silent vs. oral), reading speed, reading purpose, and individual differences.

## Conclusion

This study revisits well-established wrap-up effects and asks a set of questions on the existence, direction, and confounders to the wrap-up effect magnitudes through the lens of natural Chinese reading. We provide novel insights into the nature of wrap-up effects, challenging traditional assumptions and advancing theoretical understanding in several ways. Our main findings reveal a reversal of the classic wrap-up effect, demonstrating that sentence-final words are not necessarily subject to greater processing demands than sentence-internal words. This discovery contradicts the long-held view that sentence wrap-up inherently involves increased cognitive load at the end of clauses/sentences. Instead, the distribution of processing effort can be more context-dependently dynamic interplay between word segmentation and meaning construction, shaped by multiple linguistic factors such as reading scenario, boundary salience, wrap-up position, word type, word frequency, and word positions. Our newly-conceptualized *punctuation's dual role* underscores the importance of considering language features when theorizing about cognitive processes: in unspaced reading, sentence and clause boundaries may serve as prominent visual cues that facilitate word segmentation, support parafoveal previewing, and compensate semantic integration loads.

## Data Availability

The data and materials we utilized in the present study can be open accessed at the OSF (https://osf.io/yxkz9/?view_only=11786e2d29a84d4d811d191ebf31b8e4).

## Code availability

The present study offers an open repository at the OSF, which records the codes of data analysis (https://osf.io/yxkz9/?view_only=11786e2d29a84d4d811d191ebf31b8e4).
